# Experimental optimization for synthesis of cerium-doped titanium dioxide nanoparticles by modified sol-gel process

**DOI:** 10.1016/j.mex.2024.103071

**Published:** 2024-11-28

**Authors:** Mousab Salaheldeen Mirghani

**Affiliations:** Department of Chemical Engineering, Collage of Engineering, Najran University, Najran, Saudi Arabia

**Keywords:** Modified sol-gel method, Titanium dioxide, Photocatalysis, Cerium doping, Experimental optimization for synthesis of cerium-doped titanium dioxide nanoparticles by modified sol-gel process

## Abstract

A systematic experimental optimization procedure was developed for the synthesis of cerium-doped titanium dioxide nanoparticles (CeTNPs) based on modified sol-gel process. The nanocomposite was prepared using titanium tetraisopropoxide (TTIP) as a catalyst precursor, while utilizing the non-ionic surfactant Triton X-114 in cyclohexane as a stabilizing agent. The synthesis process was optimized by identifying the main experimental factors that affect the properties of the nanoparticles, primarily the structural phase and particle size. The synthesized samples were characterized by X-ray diffraction (XRD) for phase and size, field emission scanning electron microscope (FE-SEM) for morphology, particle size analyzer (PSA) for size distribution, and Brunaur, Emmett, and Teller (BET) for surface area and pores characteristics. The photocatalytic activity of the optimized sample was tested for the removal of methyl orange (MO) and lead (II) from aqueous solutions, and the results indicate superior performance as the catalyst uptakes were 14.8 mg/l and 11.4 mg/l for methyl orange and lead (II), respectively.

The main highlights of the proposed procedure are as follows:

• Identification of the key variables impacting the structural and morphological properties.
• Establishing the levels of each factor based on experimental findings.
• Generation of all possible combinations of factors based on ANOVA, then characterization of the synthesized material from every possible combination.

## Graphical abstract

**Figure:**
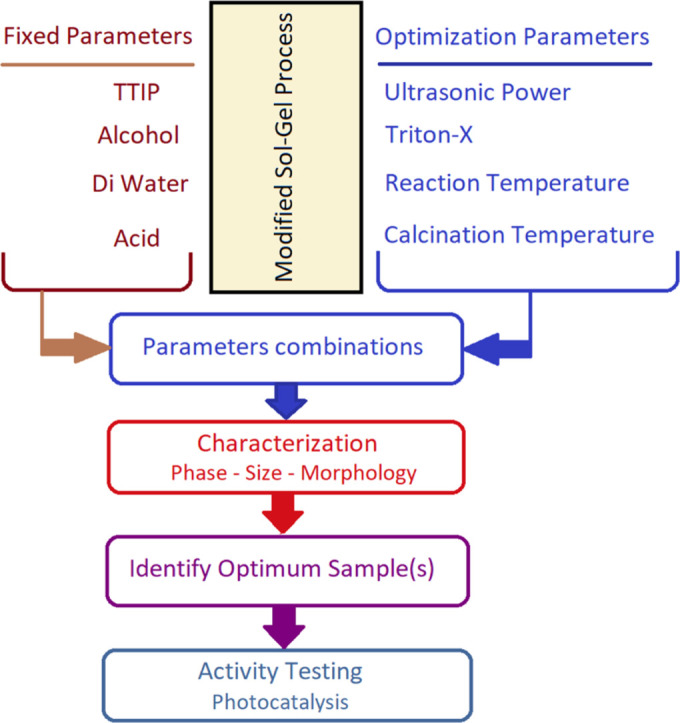

Specifications table

**Table:** 

Subject area:	Chemical Engineering
More specific subject area:	Reaction Engineering
Name of your method:	Experimental optimization for synthesis of cerium-doped titanium dioxide nanoparticles by modified sol-gel process
Name and reference of original method:	Mousab Salaheldeen Mirghani, Abdelbagi Osman, “Modified sol-gel process for synthesis of molybdenum oxide-doped titanium dioxide,” MethodsX, Volume 9, Jan. 2022, 101,742, https://doi.org/10.1016/j.mex.2022.101742
Resource availability:	Not Applicable

## Method details

Pure and metal doped titanium dioxide nanoparticles (TNPs) hold tremendous potential across diverse applications owing to their physical and chemical properties, such as non-toxicity, band gap energy, and structural stability. The synthesis of these hybrid nanocomposites has been widely investigated by different techniques, including green synthesis, chemical vapor deposition (CVD), and the sol-gel method [[1], [2], [3]]. Among these processes, the modified sol-gel process was previously developed in order to provide a relatively simple and effective procedure for the production of doped TiO2. However, a systematic experimental optimization approach is highly needed since the properties of the synthesized materials may vary significantly based on the targeted applications. This methodology provides a clear pathway for synthesis of metal doped TiO_2_ with specified and controlled properties (namely, catalyst structure and particle size) based on targeted applications, by selecting the appropriate values of experimental parameters [[4], [5], [6], [7]].

### Stage 1: Identification of optimization variable

Experimental optimization for the synthesis of pure and metal-doped titanium dioxide by the modified sol-gel method was carried out by identifying the main factors that affect the properties of the nanocrystalline catalyst, mainly the structural phase and particle size. These factors include ultrasonic power, surfactant-to-precursor molar ratio, reaction temperature, and calcination temperature. Based on the sensitivity of each factor, different levels were selected for each factor, as shown in Table 1, while keeping the amounts of catalyst precursor (TTIP), alcohol, DI water, and acid at fixed values. Factor A (ultrasonic power) was given three levels, as the process of gel formation is highly sensitive for agglomerates that may form at low power, while high power may lead to the formation of the rutile phase (a less active phase compared to anatase).

**Table 1:** Variables for experimental parameters optimization.

Factor	Parameter	Level 1	Level 2	Level 3
A	Ultrasonic power (W)	15	45	75
B	Surfactant to precursor molar ratio	1:1	1:3	–
C	Reaction temperature ( °C)	5	20	–
D	Calcination temperature ( °C)	450	750	–

### Stage 2: Experimental design and synthesis procedure

#### Materials

A precursor of titanium (IV) isopropoxide (TTIP), the non-ionic surfactant Triton X-114 in cyclohexane, hydrochloric acid, and lead (II) nitrate were purchased from Sigma-Aldrich. Cerium chloride, methyl orange (MO), and ethyl alcohol were supplied by Luba Chemicals. All materials were used without additional treatment. Deionized (DI) water was supplied from a Millipore system. All Pyrex glassware were cleaned and sterilized with soap and diluted acid, then dried at 80 °C for one hour.

#### Synthesis of Ce-Tio2 nanoparticles

In a jacketed batch reactor, a mixture of varying amounts of Triton X-114 (based on optimization run), 5 ml of titanium (IV) isopropoxide, and 12.5 ml of ethanol was prepared. The Triton X-114 enhances the distribution of Ce, by controlling doping agent within reverse micelles of the non-ionic surfactant. Another mixture containing 2.5 ml of 2000 ppm cerium solution (from cerium (III) chloride), 2 ml of sulfuric acid (0.1 M H2SO4), 5 ml of DI water, and 12.5 ml of isopropanol was prepared in a Pyrex beaker and added dropwise to the first mixture to prevent fast gel formation. The reactor temperature was adjusted to the set value. An ultrasonicator horn was mounted on top of the reactor, and the ultrasound generator was adjusted to the required power input. The reactants were stirred at 600 rpm until a milky solution (sol) was formed, then the stirrer was removed, and the setup was left at ambient conditions for gel formation. The formed gel was dried in an oven at 90 °C for 8 h, followed by calcination in a tubular furnace at varying sets of temperature (based on the optimization combination of each run as shown in Table 2). Randomly selected variations were repeated for reproducibility testing, and no considerable changes in composition and/or size were noticed (<5 %).

**Table 2:** ANOVA combinations and structural analysis results.

Run Order	Parameters Combination	Anatase to Rutile Percentage Ratio	PSA Avg. Particles Size (nm)	XRD Avg. Particles Size (nm)
R1	A1 – B1 – C1 – D1	58.1:41.9	41	45
R2	A1 – B1 – C1– D2	56.3:43.7	43	44
R3	A1 – B1 – C2– D1	55.9:44.1	40	39
R4	A1 – B1 – C2– D2	53.6:46.4	45	46
R5	A1 – B2 – C1– D1	66.2:33.8	37	41
R6	A1 – B2 – C1– D2	63.5:36.5	39	40
R7	A1 – B2 – C2– D1	64.7:35.3	32	36
R8	A1 – B2 – C2– D2	61.6:38.4	36	33
R9	A2 – B1 – C1 – D1	85.4:14.6	21	23
R10	A2 – B1 – C1– D2	78.3:21.7	23	25
R11	A2 – B1 – C2– D1	77.6:22.4	28	26
R12	A2 – B1 – C2– D2	75.1:24.9	26	24
R13	A2 – B2 – C1– D1	87.8:12.2	18	20
R14	A2 – B2 – C1– D2	82.4:17.6	23	26
R15	A2 – B2 – C2– D1	83.7:16.3	26	25
R16	A2 – B2 – C2– D2	81.9:18.1	24	27
R17	A3 – B1 – C1 – D1	55.4:44.6	44	43
R18	A3 – B1 – C1– D2	54.3:45.7	47	49
R19	A3 – B1 – C2– D1	52.1:47.9	49	47
R20	A3 – B1 – C2– D2	49.6:50.4	45	46
R21	A3 – B2 – C1– D1	58.7:41.3	37	39
R22	A3 – B2 – C1– D2	56.6:43.4	33	36
R23	A3 – B2 – C2– D1	54.2:45.8	39	41
R24	A3 – B2 – C2– D2	48.5:51.5	38	43

### Stage 3: Post-synthesis characterization

#### Structural phase and particles size analysis

**Fig. 1:**
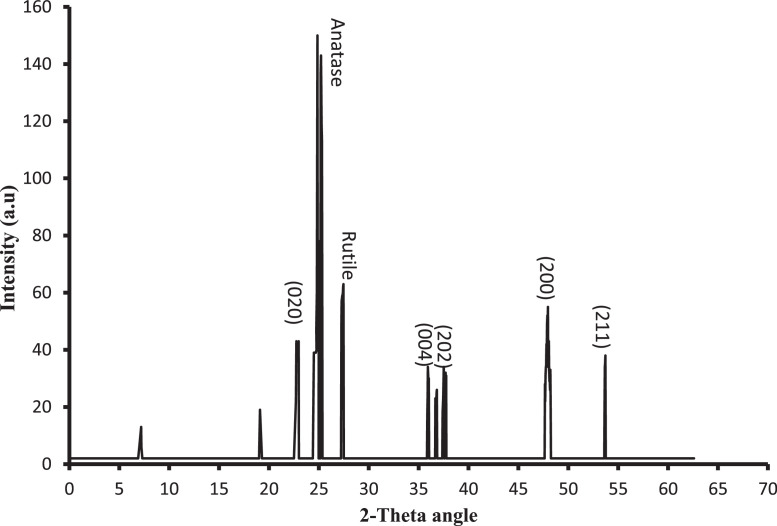
XRD analysis of the optimal sample (R13).

**Fig. 2:**
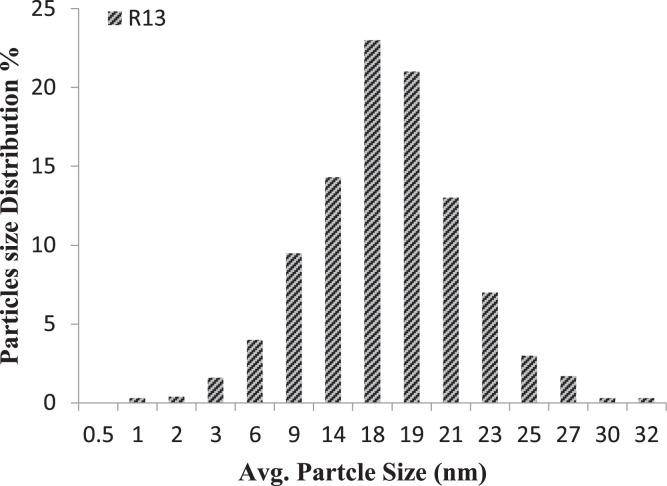
Particle size distribution of the optimal sample (R13).

**Fig. 3:**
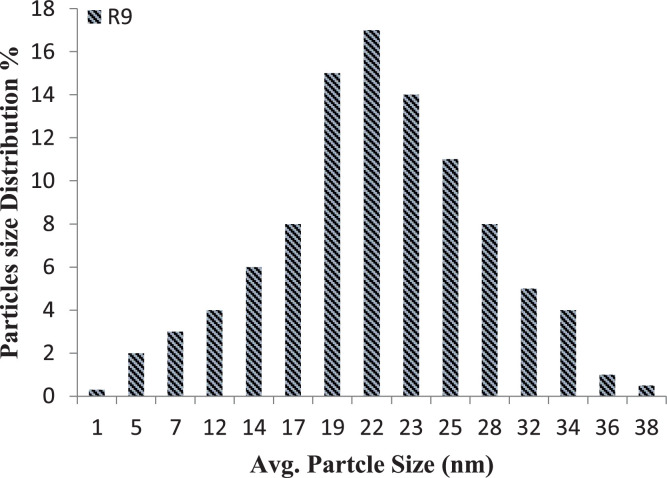
Particle size distribution of sample (R9) (near optimal).

**Fig. 4:**
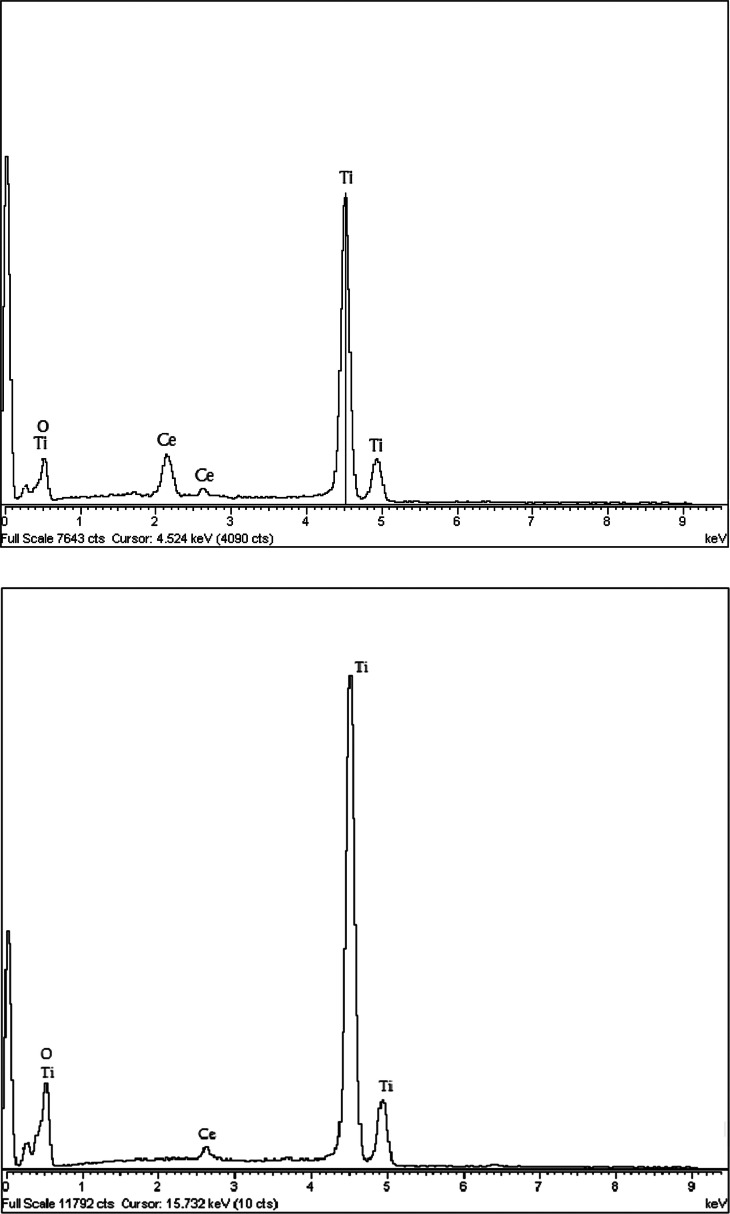
(a) EDS spectrum of favorable sample (R13). (b) EDS spectrum of less favorable sample (R20).

X-ray diffraction (XRD; Shimadzu XD3A diffractometer)analysis was carried out in order to investigate the crystalline phase (anatase to rutile ratio) and particle size based on Scherrer's formula, and the results are shown in Table 2 and Fig. 1. Additional detailed particle size analysis was also carried out using a particle size analyzer (PSA; Microtrac-Z-trac, Micromeritics-SA3500) to further confirm the average particle size for each sample, as shown in Table 2, as well as provide detailed insight into size distribution, as shown in Fig. 2, Fig. 3 (for optimal combinations). Energy dispersive X-ray spectroscopy (EDS; OXFORD INCAx-SIGHT) analysis was performed to confirm the composition of all synthesized samples, and the results showed the presence of Ce in all doped TiO2 variationes. Fig. 4a and b show the EDS spectrums for favorable sample (R13) and less favorable sample (R20), respectively, as a confirmation of composition.

## Method validation

### Morphological analysis

**Fig. 5:**
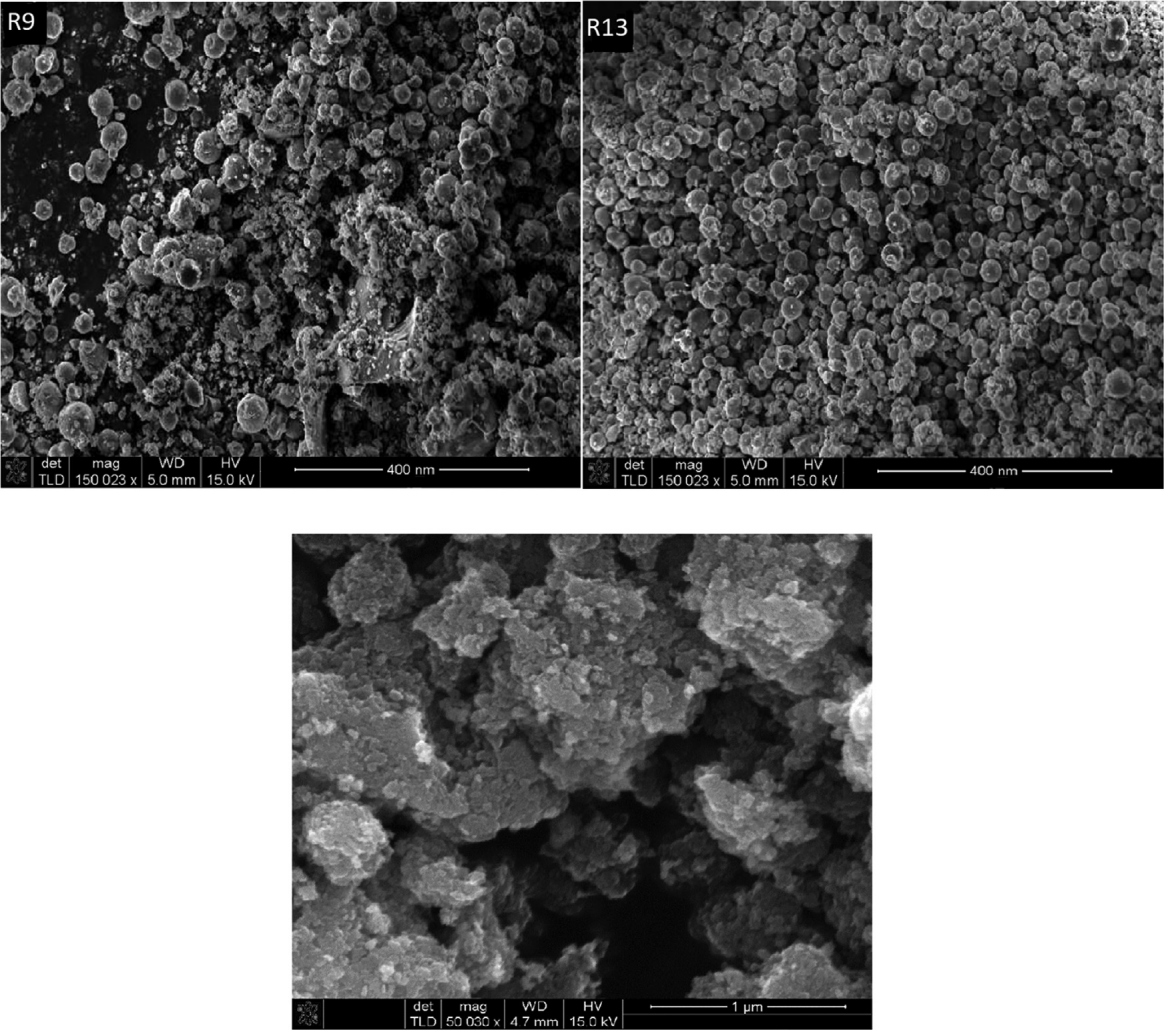
(a) FE-SEM images of samples R9 (left) and R13 (right). (b) FE-SEM images of sub-optimal sample (R20).

Field emission scanning electron microscope (FE-SEM; FEI-Nova-Nano-FE-SEM) analysis was carried out for the optimized samples (R9 and R13), in addition to one sub-optimal sample (R20), and the resulting images are shown in Fig. 5(a and b). The results show spherical crystalline nanoparticles for sample R13 (right), while the closed-to-optimal sample (R9) has agglomerates, which can be attributed to the lower surfactant-to-precursor molar ratio.

### Surface area and porosity

Brunaur, Emmett, and Teller (BET; Quanta-chrome-NovaTech: M40) analysis for the optimum runs (samples R9 and R13) was carried out in order to investigate the surface area and pore structure (volume and width) as shown in Table 3. The results show a relatively high surface area, as calculated by both theBET and Langmuir models, while the measured average pore width falls in the mesoporous type range (2–50 nm).

**Table 3:** BET analysis of the optimal samples, R9 and R13.

Property	Sample No R9	Sample No R13
BET surface area (m²/g)	103.8	107.2
Langmuir surface area (m²/g)	116.5	129.3
Average pore volume at p/p° = 0.141(cm³/g)	0.037	0.046
BET average pore width (nm)	7.58	6.91

### Photocatalytic applications experiments

**Fig. 6:**
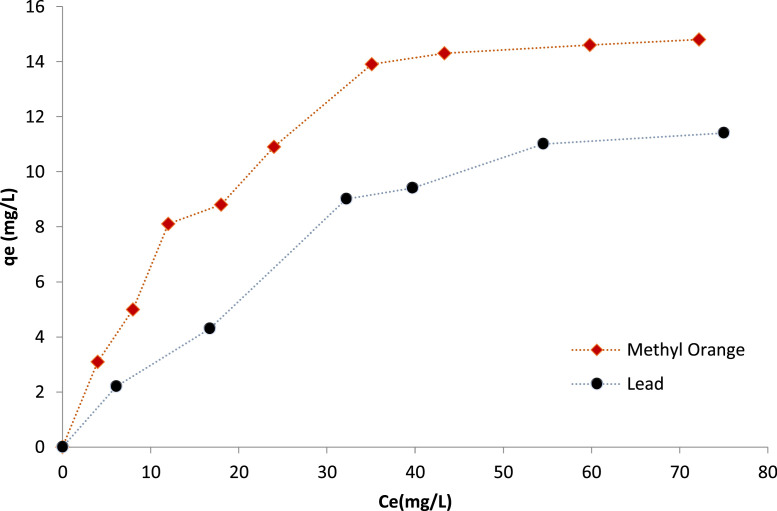
Removal of methyl orange and lead (II) by CeTNP (sample R13).

The photocatalytic activity of the synthetic Ce-TiO_2_ (R13) nanocatalyst was tested by the degradation of MO in aqueous solutions. In these experiments, solutions of MO with concentrations ranging from 10 ppm to 100 ppm were prepared from a 200 ppm stock solution. Each solution was equally divided into two beakers, and 0.2 g of catalyst was added to one solution, while keeping the other as blank. All solutions were left under UV exposure (intensity of 1200 mW m-2, 254 nm) for 15 h to reach equilibrium, then the final concentration of MO in each solution was measured using UV–vis (UV–Vis; Agilent Cary-3500), and the differences in equilibrium concentrations between the catalyst containing solutions and the blanks were reported. A similar procedure was followed for the reduction of Pb^2+^, where an atomic absorption spectrometer (AAS; Thermo-Scientific-i-CAP4200) was used to measure the concentration of lead in each solution. The results shown in Fig. 6 indicate significant catalytic uptake of 14.8 mg/l and 11.4 mg/l for MO and Pb^2+^, respectively, which proves the effectiveness of this procedure for nanocomposites synthesis for environmental remediation applications [[8], [9], [10]]. Applications of metal doped-TiO_2_ for reduction of heavy metals and removal of dyes from aqueous solutions generally requires a higher ratio of anatase to rutile phases, and smaller particle size to maximize the catalyst surface area, so for these type of applications, samples (R13), (R9), and (R14) may be considered more favorable. Other variations can be considered for different applications, such as catalytic promoters, based on the required phase ratio and size.

**Fig. 7:**
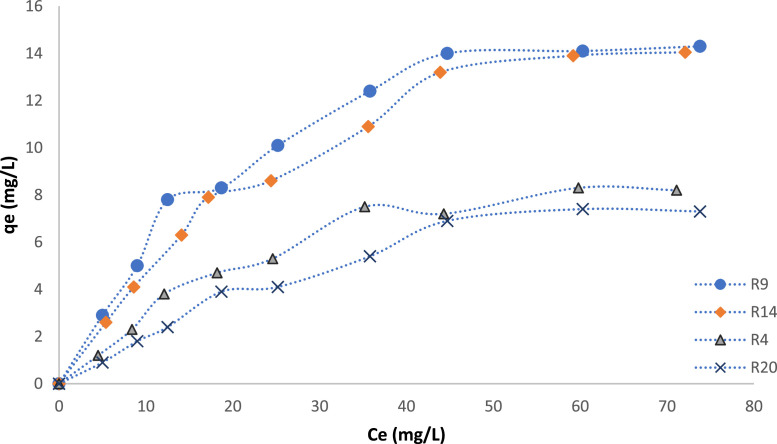
Removal of methyl orange by CeTNP (samples R4, R9, R14, and R20).

Further investigations for the photocatalytic activity of the more favorable samples (R9 and R14), and the least favorable samples (R4 and R20) were caried out by the degradation of methyl orange, and the results shown in Fig. 7 indicate significant drop in catalyst uptake as the catalyst size and anatase to rutile ratios were increased.

## CRediT author statement

**Mousab Salaheldeen Mirghani:** Conceptualization, Methodology,Validation, Formal analysis, Writing, Reviewing,and Editing.

## Ethics statements

Not applicable.

## Declaration of competing interest

The authors declare that they have no known competing financial interests or personal relationships that could have appeared to influence the work reported in this paper.

## Data Availability

Data will be made available on request.

## References

[1] Mirghani M.S., Osman A. (Jan. 2022). Modified sol-gel process for synthesis of molybdenum oxide-doped titanium dioxide. MethodsX..

[2] Susan Punnoose M., Bijimol D., Mathew B. (2021). Microwave assisted green synthesis of gold nanoparticles for catalytic degradation of environmental pollutants. Environ. Nanotechnol. Monit. Manage.

[3] Deepika M.Dixit, Singh H., Attia M.S., Amin M.A. (Jan. 2021). Recent innovations in properties of nanostructured glasses and composites. J. Exp. Nanosci..

[4] Le A.T., Tan Z.-H., Sivakumar R., Pung S.-Y. (2021). Predicting the photocatalytic performance of metal/metal oxide coupled TiO2 particles using Response Surface Methodology (RSM). Mater. Chem. Phys..

[5] Mirghani M.S. (Jan. 2021). Vanadium doped titania nanoparticles for photocatalytic removal of heavy metals from aqueous solutions. J. Exp. Nanosci..

[6] Padmini M., Balaganapathi T., Thilakan P. (2021). Mesoporous rutile TiO2: synthesis, characterization and photocatalytic performance studies. Mater. Res. Bull..

[7] Ruzimuradov Olim (2023). Structural and optical properties of sol-gel synthesized TiO2 nanocrystals: effect of Ni and Cr (co)doping. Opt. Mater..

[8] Pandiyaraj V., Murmu A., Pandy S.K., Sevanan M., Arjunan S. (2021). Metal nanoparticles and its application on phenolic and heavy metal pollutants. Phys. Sci. Rev..

[9] Mirghani M., Al-Mubaiyedh U.A., Nasser M.S., Shawabkeh R. (2015). Experimental study and modeling of photocatalytic reduction of Pb2+ by WO3/TiO2 nanoparticles. Sep. Purif. Technol..

[10] Zhang X., Chen W.-F., Bahmanrokh G. (2020). Synthesis of V- and Mo-doped/codoped TiO2 powders for photocatalytic degradation of methylene blue. Nano-Struct. Nano-Objects.

